# Eczema

**Published:** 1890-04

**Authors:** 


### ECZEMA.

Is one of the many eruptive diseases of the skin. The blood-vessels of the parts affected are in a state of congestion, accompanied by itching, smarting, and exudation of serum, or watery portion of the blood. The disease varies greatly in severity and extent, as well as in its course and character. Its simplest form is a mere redness, perhaps on the eyelids or behind the ears, or near the joints. Sometimes there are pimples, either on the affected spots, or around them, or more or less diffused over the body. Sometimes vesicles—water-bladders—are formed by the exudation of serum beneath the skin, the special seat being the back of the hand, or the front and sides of the fingers. In a few days the serum may be absorbed ; the swelling subsides, the cuticle dries up and comes off, and the skin either returns to its normal condition, or the cuticle is thrown off in scales. In another variety there is intense redness, profuse exudation, and the formation of a thick crust, through fissures in which a mucous pus exudes. The worst period of eczema, when chronic, may be characterized by a coming off of the cuticle in thin, fine scales, or by a tendency of the skin to chap and crack ; sometimes the mere stretching of the fingers will cause it to break. In some cases the skin becomes as hard and tough as leather, with an inclination to itch and throw off dry and scaly scurf ; more rarely it is rough like an old wart, in which case the itching is generally very severe. As a rule, the eczema occurs in limited patches, but occasionally it spreads over a large part of the trunk or limb. There is hardly any part of the body which it may not attack. It is not contagious. The disease may result from a condition of the body, —from constitutional debility, or temporary derangement of the nervous or digestive organs, or even from unsuitable or insufficient food, or it may have an external exciting cause—cold or heat in excess, insufficient clothing, or garments that irritate the skin. The treatment must be first directed against that which causes the condition of which the eczema is only a symptom. At the same time careful local treatment will be necessary. But no general directions can be given suited to so variable a disease. A skilled physician should have charge of the case.

